# Empirical Bayes analysis of single nucleotide polymorphisms

**DOI:** 10.1186/1471-2105-9-144

**Published:** 2008-03-06

**Authors:** Holger Schwender, Katja Ickstadt

**Affiliations:** 1 Collaborative Research Center 475, Faculty of Statistics, Dortmund University of Technology, 44221 Dortmund, Germany

## Abstract

**Background:**

An important goal of whole-genome studies concerned with single nucleotide polymorphisms (SNPs) is the identification of SNPs associated with a covariate of interest such as the case-control status or the type of cancer. Since these studies often comprise the genotypes of hundreds of thousands of SNPs, methods are required that can cope with the corresponding multiple testing problem. For the analysis of gene expression data, approaches such as the empirical Bayes analysis of microarrays have been developed particularly for the detection of genes associated with the response. However, the empirical Bayes analysis of microarrays has only been suggested for binary responses when considering expression values, i.e. continuous predictors.

**Results:**

In this paper, we propose a modification of this empirical Bayes analysis that can be used to analyze high-dimensional categorical SNP data. This approach along with a generalized version of the original empirical Bayes method are available in the R package siggenes version 1.10.0 and later that can be downloaded from  .

**Conclusion:**

As applications to two subsets of the HapMap data show, the empirical Bayes analysis of microarrays cannot only be used to analyze continuous gene expression data, but also be applied to categorical SNP data, where the response is not restricted to be binary. In association studies in which typically several ten to a few hundred SNPs are considered, our approach can furthermore be employed to test interactions of SNPs. Moreover, the posterior probabilities resulting from the empirical Bayes analysis of (prespecified) interactions/genotypes can also be used to quantify the importance of these interactions.

## Background

Whole-genome experiments comprise data of hundreds of thousands of single nucleotide polymorphisms (SNPs), where a SNP is the most common type of genetic variations that occurs when at a single base pair position different base alternatives exist in a population. SNPs are typically biallelic. Therefore, SNPs can be interpreted as categorical variables having three realizations: the homozygous reference genotype (if both chromosomes show the more frequent variant), the heterozygous genotype (if one chromosome shows the more frequent, and the other the less frequent variant), and the homozygous variant genotype (if both bases explaining the SNP are of the less frequent variant).

Since SNPs can alter the risk for developing a disease, an important goal in studies concerned with SNPs is the identification of the SNPs that show a distribution of the genotypes that differs substantially between different groups (e.g., cancer vs. non-cancer). Detecting such SNPs requires methods that can cope with this vast multiple testing problem in which hundreds of thousands of hypotheses are tested simultaneously. Naturally, the value of a statistic appropriate for the considered testing situation and the corresponding *p*-value are computed for each variable, where in the case of SNPs Pearson's *χ*^2^-statistic is an appropriate test score. These raw *p*-values are then adjusted for multiple comparisons such that a Type I error rate is strongly controlled at a prespecified level of significance *α*.

The classical example for a Type I error rate is the family-wise error rate

FWER = Prob(*V *≥ 1),

where *V *is the number of false positives, i.e. the number of rejected null hypotheses that are actually true – or in biological terms, the number of SNPs found by the procedure to differ between groups that actually do not differ between the groups. This error rate is strongly controlled at a level *α *so that Prob(*V *≥ 1) ≤ *α *by approaches such as the Bonferroni correction or the procedures of Westfall and Young [1]. An overview on such methods is given in [2]. In [3], procedures for controlling this and other error rates are compared in an application to gene expression data.

In classical multiple testing situations in which rarely more than 20 hypotheses are tested simultaneously, it is reasonable to keep down the probability of one or more false positives. However, in the analysis of data from whole-genome studies, hundreds of thousands of SNPs are considered simultaneously. Moreover, a few false positives are acceptable in such experiments as long as their number is small in proportion to the total number *R *of rejected null hypotheses, i.e. identified SNPs. This situation for which the family-wise error rate might be too conservative is thus similar to the multiple testing problem in studies concerned with gene expression data. In the analysis of such DNA microarray data, another error rate, namely the false discovery rate

$$\text{FDR}=\{\begin{array}{ll} \text{E}(V/R), & \text{if }R>0\\ 0, & \text{if }R=0 \end{array}$$

proposed by Benjamini and Hochberg [4], has hence become popular which in turn is a reasonable choice in the analysis of high-dimensional SNP data.

Apart from adjusting *p*-values, there also exist other approaches for adjusting for multiple comparisons such as the significance analysis of microarrays (SAM [5]) and the empirical Bayes analysis of microarrays (EBAM [6]) that have been developed particularly for the analysis of gene expression data.

In the original versions of both SAM and EBAM, a moderated *t*-statistic is computed. In SAM, the observed values of this test score are then plotted against the values of the statistic expected under the null hypothesis of no difference between the two groups, and a gene is called differentially expressed if the point representing this gene in this Quantile-Quantile plot is far away from the diagonal. In EBAM, the density *f *of the observed values *z *of the moderated *t*-statistic is modeled by a mixture of the density *f*_1 _of the differentially expressed genes and the density *f*_0 _of the not differentially expressed genes, i.e. by

*f*(*z*) = *π*_0_*f*_0_(*z*) + *π*_1_*f*_1_(*z*),

where *π*_1 _and *π*_0 _= 1 - *π*_1 _are the prior probabilities that a gene is differentially expressed or not, respectively. Following Efron et al. [6], a gene having a *z*-value of z* is detected to be differentially expressed if the posterior probability

$$p_{1}(z^{*})=1-\pi_{0}\frac{f_{0}(z^{*})}{f(z^{*})}$$

for being differentially expressed is larger than or equal to 0.9.

In [7], a generalized version of the SAM algorithm is presented, whereas in [8,9] SAM is adapted for categorical data such as SNP data.

In the following section, we first present a generalized EBAM algorithm. Then, we propose an adaption of EBAM enabling the analysis of categorical data. As computing the values of the test statistic for all SNPs individually would be very time-consuming, we further suggest an approach based on matrix algebra that allows to compute all values simultaneously. Afterwards, EBAM for categorical data is applied, on the one hand, to two subsets of the high-dimensional SNP data from the HapMap project [10], and on the other hand, to simulated data that mimic data from a typical association study in which several ten SNPs are considered. In the latter application, it is also shown how EBAM can be applied to identify SNP interactions associated with the response, and how it can be used to specify the importance of prespecified SNP interactions.

## Methods

### Generalized EBAM algorithm

In Algorithm 1, a generalized version of the empirical Bayes analysis of microarrays (EBAM [6]) is presented. This algorithm makes use of the fact that for a given rejection region Γ, the FDR can be estimated by

$$\bar{\text{FDR}}(\Gamma )=\pi_{0}\frac{{\text{E}}_{{\text{H}}_{0}}\left(\#\{Z_{i}\in \Gamma \}\right)}{\max\left\{\#\{z_{i}\in \Gamma \},1\right\}},$$

where *z*_*i *_is the observed value of the test statistic *Z*_*i *_for variable *i *= 1 ⋯ *m*, *π*_0 _is the prior probability that a gene is not differentially expressed – or more generally, that a variable is not associated with the response – and ${\text{E}}_{{\text{H}}_{0}}$ (#{*Z*_*i *_∈ Γ}) is the number of values expected under the null hypothesis to fall into Γ [11].

Several procedures have been suggested to estimate the prior probability *π*_0_[6,11,12]. Efron et al. [6], e.g., propose to use a narrow interval A around *z *= 0, and to estimate *π*_0 _by the ratio of the number of observed *z*-values in A to the number of *z*-values that are expected under the null hypothesis to fall into A. However, the narrower A, the more instable is this estimate. To stabilize this estimate, we use the procedure of Storey and Tibshirani [12] in which a natural cubic spline *h *with three degrees of freedom is fitted through the data points

$$\begin{array}{cc} \left(\lambda ,\frac{\#\left\{z_{i}\in \Lambda \right\}}{\left(1-\lambda \right)m}\right), & \lambda =0.00,0.01,...,0.95, \end{array}$$

where

$$\Lambda =\{\begin{array}{ll} {}[0,q_{1-\lambda }), & \text{if }\Gamma \text{ is one-sided}\\ (q_{\lambda /2},q_{1-\lambda /2}), & \text{if }\Gamma \text{ is two-sided} \end{array}$$

and *q*_*λ *_denotes the *λ *quantile of the (estimated) null distribution. The estimate of *π*_0 _is then given by

$${\hat{\pi }}_{0}=\min\left\{h(1),1\right\}.$$

### Algorithm 1 (Generalized EBAM Procedure)

Let **X **be an *m *× *n *matrix comprising the values of *m *variables and *n *observations, **y **be a vector of length *n *composed of the values of the response for the *n *observations, and *B *be the number of permutations.

1. For each variable *i *= 1, ..., *m*, compute the value *z*_*i *_of a statistic appropriate for testing if the values of this variable are associated with the response.

2. If the null density *f*_0_, is known, use a density estimation procedure to obtain $\hat{f}$ and compute $\hat{\phi }=f_{0}/\hat{f}$. Otherwise, estimate the ratio *ϕ *= *f*_0_/*f *directly by

(a) determining the *m *permuted *z*-values *z*_*ib *_for each permutation *b *= 1, ..., *B *of the *n *values of the response,

(b) binning the *m *observed and *mB *permuted *z*-values into an appropriate number of intervals,

(c) fitting a logistic regression model with repeated observations through these intervals using an appropriate regression function.

3. Estimate *π*_0 _by the procedure of Storey and Tibshirani [12].

4. For each variable *i*, compute the posterior probability ${\hat{p}}_{1}(z_{i})=1-{\hat{\pi }}_{0}\hat{\phi }(z_{i})$

5. Order the observed *z*-values to obtain *z*_(1) _≤ ... ≤ *z*_(*m*)_, and set $i_{0}=\sum_{i=1}^{m}I(z_{\left(i\right)}<0)+1$

6. For a prespecified probability Δ or a set of appropriate values for Δ,

(a) set $i_{1}=\max_{i\geq i_{0}}\left\{i:{\hat{p}}_{1}(z_{\left(i\right)})<\Delta \right\}+1$, and compute the upper cut-off *c*_*U *_by

$$c_{U}=\{\begin{array}{ll} z_{\left(i_{1}\right)}, & \text{if }i_{1}\leq m\\ \infty & \text{otherwise} \end{array},$$

(b) set $i_{2}=\min_{i<i_{0}}\left\{i:{\hat{p}}_{1}(z_{\left(i\right)})<\Delta \right\}-1$, and compute the lower cut-off *c*_*L *_by

$$c_{L}=\{\begin{array}{ll} z_{\left(i_{2}\right)}, & \text{if }i_{0}>1\text{ and }i_{2}\geq 1\\ -\infty & \text{otherwise} \end{array},$$

(c) call all variables *i *with $z_{i}\notin \Gamma_{\Delta }^{C}$ significant, where $\Gamma_{\Delta }^{C}=(c_{L},c_{U})$ denotes the complement of the rejection region Γ_Δ_,

(d) estimate the FDR of Γ_Δ _by

$$\bar{\text{FDR}}(\Gamma_{\Delta })={\hat{\pi }}_{0}\frac{\alpha m}{\max\left\{\#\left\{z_{i}\in \Gamma_{\Delta }\right\},1\right\}},$$

where

$$\alpha =\{\begin{array}{ll} 1-\int_{c_{L}}^{c_{U}}f_{0}(z)\text{d}z, & \text{if }f_{0}\text{ is known}\\ \frac{\#\left\{z_{ib}\in \Gamma_{\Delta }\right\}}{mB} & \text{otherwise} \end{array}.$$

The original version of EBAM is of course a special case of Algorithm 1: Efron et al. [6] compute the moderated *t*-statistic

(1) $$z_{i}=\frac{d_{i}}{a_{0}+s_{i}}$$

for each gene *i *= 1, ..., *m*, where *d*_*i *_is the difference of the groupwise mean expression values and *s*_*i *_is the corresponding standard deviation such that *d*_*i*_/*s*_*i *_is the ordinary *t*-statistic. The fudge factor *a*_0 _is computed by the quantile of the *m *standard deviations that leads to the largest number of genes called differentially expressed in a standardized EBAM analysis (see [6] for details on this standardized analysis). Since the null distribution of (1) is unknown, the response is permuted repeatedly to generate *mB *permuted *z*-values. Efron et al. [6] then bin the *m *observed and *mB *permuted *z*-values into 139 intervals. Treating the observed scores as successes and the permuted values as failures, a logistic regression model is fitted through the binned data points using a natural cubic spline with five degrees of freedom as regression function. For details on this logistic regression, see Remark (D) in [6].

Algorithm 1 also comprises the approach used by Efron and Tibshirani [13] to test two-group gene expression data with Wilcoxon rank statistics.

The main difference between Algorithm 1 and the original version of EBAM is that Efron et al. [6] call all genes differentially expressed that have a posterior probability larger than or equal to Δ = 0.9, whereas we only call a variable *i *with $\hat{p}$_1_(*z*_*i*_) ≥ Δ significant if there is no other variable with a more extreme *z*-value (a larger *z*-value if *z*_*i *_> 0, or a smaller *z*-value if *z*_*i *_< 0) that has a posterior probability less than Δ. This approach that is comparable to the proceeding in SAM, therefore, ensures that all variables with a *z*-value exceeding some threshold are called significant, whereas in the original version of EBAM it might happen that a variable is not called significant, even though it has a more extreme *z*-value than some of the identified variables.

Another difference is that Efron et al. [6] consider one fixed posterior probability, namely Δ = 0.9, for calling genes differentially expressed, whereas we allow both to prespecify one probability Δ and to consider a set of reasonable values for Δ. The latter again is similar to the SAM procedure in which the number of genes called differentially expressed and the estimated FDR is determined for several values of the SAM threshold, and then the value is chosen that provides the best balance between the number of identified genes and the estimated FDR. This approach can be helpful when the detection of interesting variables is just an intermediate aim, and the actual goal of the analysis is, e.g., the construction of a classification rule. In such a case, prespecifying the value of Δ might work poorly, as this might lead to either a too small number of identified variables, or a too high FDR. For an example of this proceeding in the context of the empirical Bayes analysis, see the application of EBAM for categorical data to the HapMap data set.

### EBAM for categorical data

We now assume that our data consist of *m *categorical variables each exhibiting *C *levels denoted by 1, ..., *C*, and *n *observations each belonging to one of *R *groups denoted by 1, ..., *R*. If these variables are SNPs, *C *= 3.

A statistic appropriate for testing each of the *m *categorical variables if its null distribution differs between the *R *groups is Pearson's *χ*^2^-statistic

(2) $$\chi^{2}=\sum_{r=1}^{R}\sum_{c=1}^{C}\frac{{\left(n_{rc}-{\tilde{n}}_{rc}\right)}^{2}}{{\tilde{n}}_{rc}}=\sum_{r=1}^{R}\sum_{c=1}^{C}\frac{n_{rc}^{2}}{{\tilde{n}}_{rc}}-n,$$

where *n*_*rc *_and $\tilde{n}$_*rc *_are the observed number of observations and the number of observations expected under the null hypothesis in group *r *= 1, ..., *R*, respectively, showing level *c *= 1, ..., *C*.

Since the small denominator problem [5,6,14], which is the reason for adding the fudge factor *a*_0 _to the denominator of the ordinary *t*-statistic in (1), does not show up in this case, it is not necessary to add a fudge factor to the denominator of (2). Therefore, Algorithm 1 can be applied to SNPs – or to any other type of (genetic) categorical data – by employing Pearson's *χ*^2^-statistic as test score.

In EBAM, it is assumed that all variables follow the same null distribution. In the permutation based approach of Algorithm 1, this, e.g., means that not only the *B *permuted *z*-values corresponding to a particular variable, but all *mB *permutations of all *m *variables are considered in the estimation of the null distribution of this variable. Normally, this is an advantage in the analysis of high-dimensional data [6,15]. In the analysis of categorical data, this, however, might lead to a loss of a large number of variables, as only variables showing the same number of levels can be considered together in an EBAM analysis.

#### Approximation to *χ*^2^-distribution

Since the null distribution of (2) can be approximated by a *χ*^2^-distribution with (*R *- 1)(*C *- 1) degrees of freedom, only the density *f *of the observed test statistics needs to be estimated. This can be done by applying a (non-parametric) kernel density estimator to the observed *z*-values [16]. However, the standard kernels are typically symmetric such that negative values of *z *will have a positive estimated density, even though *f*(*z*) = 0 for *z *< 0. A solution to this problem is to use asymmetric kernels that only give non-negative values of *z *a positive density [17,18]. Another solution, which we will use, is a semi-parametric method proposed by Efron and Tibshirani [19].

In the first step of this procedure, a histogram of the observed *z*-values is generated. To obtain a reasonable number of bins for the histogram, we employ the one-level bin width estimator of Wand [20]. Although other bin width estimators such as the approaches of Scott [21] or of Freedman and Diaconis [22] lead to different bin widths, the densities resulting from the method of Efron and Tibshirani [19] are virtually identical. The approach of Sturges [23], however, which is, e.g., the default method for estimating the number of bins in the R function hist, typically leads to a much too small number of intervals when considering large numbers of observations [24], and is therefore an inappropriate procedure in our application.

**Figure 1:**
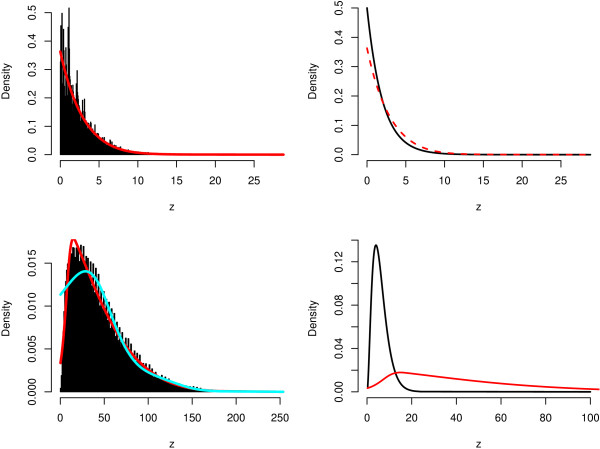
**Densities of the test scores in the analyses of the HapMap data**. On the left hand side, the histograms and the estimated densities (marked by red lines) of the values of Pearson's *χ*^2^-statistic of the SNPs from the two subsets of the HapMap data (upper panel: JPT vs. CHB, lower panel: all four HapMap populations) are shown. The cyan line marks the estimated density when the inner knots are centered around the median in the natural cubic spline used in the density estimation. On the right hand side, the estimated densities (again, marked by red lines) and the corresponding null densities (black lines) are displayed.

In the second step of the procedure of Efron and Tibshirani [19], a Poisson regression model is fitted in which the midpoints of the bins are used as explanatory variables, and the numbers of observations in the intervals are the values of the response. As most of the SNPs are assumed to show the same distribution in the different groups, the density *f *of the observed *z*-values typically looks similar to the null density *f*_0_, but has a heavier right tail (see Figure 1). We therefore use a natural cubic spline with three degrees of freedom as regression function if (*R *- 1)(*C *- 1) ≤ 2. For (*R *- 1)(*C *- 1) ≥ 3, a natural cubic spline with five degrees of freedom would be a reasonable regression function. However, in functions such as the R function ns for generating the basis matrix of the spline, the inner knots by default are given by the 20%, 40%, 60%, and 80% quantile of the midpoints of the bins. These inner knots work well for symmetric densities. But the *χ*^2^-distribution is asymmetric – in particular for a small value of the degrees of freedom. If (*R *- 1)(*C *- 1) ≥ 3, we hence specify the inner knots directly by centering them around the mode and not around the median. The inner knots are thus given by the 0.4*q*_M_, 0.8*q*_M_, 1 - 0.8(1 - *q*_M_), and 1 - 0.4(1 - *q*_M_) quantile of the midpoints, where *q*_M_ is the quantile of the midpoints that corresponds to the mode estimated by the midpoint of the bin of the histogram containing the most observations. If there is more than one bin showing the largest number of observations, then the smallest of the corresponding midpoints is used as estimate. Other mode estimators such as the half-range mode [25,26] might lead to better estimates than this ad hoc methods, but the estimation of *f *is typically only slightly influenced by the choice of the mode estimator.

**Figure 2:**
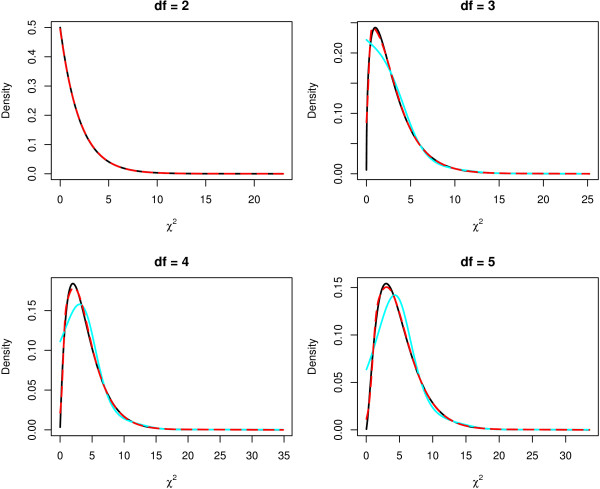
**Estimating the density of the *χ*^2^-distribution**. For different degrees of freedom, the true (black line) and the estimated density (red line) of the *χ*^2^-distribution are shown, where the density is estimated by applying the procedure of Efron and Tibshirani [19] to 100,000 values randomly drawn from the *χ*^2^-distribution. The cyan line marks the estimated density when the inner knots of the natural cubic spline are centered around the median in the *df *≥ 3 case.

In Figure 2, the estimated densities of four *χ*^2^-distributions with different degrees of freedom resulting from the application of this procedure to 100,000 values randomly drawn from the respective *χ*^2^-distribution are displayed, where the inner knots are centered, on the one hand, around the mode (red lines), and on the other hand, around the median (cyan lines). This figure reveals that the former leads to a better estimation than using the standard inner knots. In fact, the densities estimated using the former approach are very similar to the true densities.

Having estimated *f*, $\hat{\phi }=f_{0}/\hat{f}$ is determined, and the remaining steps 3 to 6 of Algorithm 1 are processed.

#### Permutation based estimation of the null density

If the assumptions for the approximation to the *χ*^2^-distribution are not met [27], the null density *f*_0 _also has to be estimated. In this case, we calculate the ratio $\hat{\phi }$ directly by permuting the group labels *B *times, computing the *mB *permuted *z*-values, dividing these scores and the *m *observed *z*-values into intervals, and fitting a logistic regression model through the binned data points. Similar to the application of the procedure of Efron and Tibshirani [19] (see previous section), the estimation of *ϕ *does not depend on the number of intervals used in the binning as long as this number is not too small or too large. We therefore follow Efron et al. [6], and split the observed and permuted *z*-values into 139 intervals. Since the rejection region is one-sided when considering Pearson's *χ*^2^-statistic as test score, a natural cubic spline with three degrees of freedom is used as regression function.

### Implementation

Whole-genome studies comprise the genotypes of hundreds of thousands of SNPs for each of which the value of Pearson's *χ*^2^-statistic (2) has to be computed. Since calculating these values one-by-one is very time-consuming, we employ matrix algebra for determining all the scores simultaneously.

Assume that we have given an *m *× *n *matrix **X **in which each row corresponds to a categorical variable exhibiting the levels 1, ..., *C*, and a vector **y** comprising the group labels 1, ..., *R *of the *n *observations represented by the columns of **X**.

Firstly, *C m *× *n *indicator matrices **X**^(*c*) ^for the *C *levels are constructed by setting the elements of these matrices to

$$x_{ij}^{\left(c\right)}=I(x_{ij}=c)=\{\begin{array}{cc} 1, & \text{if }x_{ij}=c\\ 0 & \text{otherwise} \end{array},$$

*i *= 1, ..., *m*, *j *= 1, ..., *n*. Furthermore, an *n *× *R *matrix **Y **with entries *y*_*jr *_= *I*(*y*_*j *_= *r*) is built in which each column represents one of the *R *group labels. Then, we set

**N**^(*c*) ^= **X**^(*c*)^**Y**

and

$${\tilde{N}}^{\left(c\right)}=\frac{1}{n}X^{\left(c\right)}1_{n}{1^{\prime }}_{n}Y,$$

*c *= 1, ..., *C*, where **1**_*n *_is a vector of length *n *consisting only of ones, so that the *i*th column and *r*th row of the *m *× *R *matrices **N**^(*c*) ^and $\tilde{N}$^(*c*) ^comprise the observed and the expected number of observations, respectively, that belong to the *r*th group and show the *c*th level at the *i*th variable. Afterwards, the *m *× *R *matrices

$$\begin{array}{cc} S^{\left(c\right)}=\frac{N^{\left(c\right)}*N^{\left(c\right)}}{{\tilde{N}}^{\left(c\right)}}, & c=1,...,C \end{array},$$

are determined by elementwise matrix calculation, i.e. by setting

$$s_{ir}^{\left(c\right)}=\frac{n_{ir}^{\left(c\right)}\cdot n_{ir}^{\left(c\right)}}{{\tilde{n}}_{ir}^{\left(c\right)}}.$$

Finally, the vector **z **comprising the value of Pearson's *χ*^2^-statistic for each of the *m *variables is given by

$$z=\sum_{c=1}^{C}S^{\left(c\right)}1_{R}-n.$$

If the permutation based version of EBAM for categorical data is used, then not "just" *m*, but *m*(*B *+ 1) *z*-values have to be computed. Again, matrix algebra can help to speed up computation by considering all *B *permutations at once, or – if the number of variables or permutations is too large – subsets of the *B *permutations.

For this, suppose that **L **is a *B *× *n *matrix in which each row corresponds to one of the *B *permutations of the *n *group labels. If the *B *× *n *indicator matrices **L**^(*r*)^, *r *= 1, ..., *R*, are defined analogously to **X**^(*c*)^, then the *m *× *B *matrix **Z**^0 ^= {*z*_*ib*_} containing the *mB *permuted *z*-values can be determined by

$$Z^{0}=\sum_{c=1}^{C}\sum_{r=1}^{R}\frac{\left(X^{\left(c\right)}L^{(r{)}^{\prime }}\right)*\left(X^{\left(c\right)}L^{(r{)}^{\prime }}\right)}{{\tilde{n}}_{r}^{\left(c\right)}⊗{1^{\prime }}_{B}}-n,$$

where ${\tilde{n}}_{r}^{\left(c\right)}$ is the *r*th column of $\tilde{N}$, ⊗ is the symbol for the Kronecker product, and * and the fraction line denote elementwise matrix calculation.

### Processing time

To evaluate how much the matrix calculation procedure presented in the previous section can speed up the computation in comparison to an individual determination of Pearson's *χ*^2^-statistic, both approaches are applied to several numbers of variables. In Table 1, the resulting processing times are summarized. This table shows that employing matrix algebra leads to an immense reduction of time needed for computation – in particular if the number *m *of variables is large. If, e.g., 100,000 variables are considered, it takes just 6.2 seconds to determine the values of Pearson's *χ*^2^-statistic when employing matrix calculation, but more than 4.5 minutes when calculating the values one-by-one.

**Table 1:** Comparison of computation times (in seconds) on an AMD Athlon XP 3000+ machine with one GB of RAM for both the matrix algebra based calculation and the individual determination of the values of Pearson's *χ*^2^-statistic for different numbers of variables and observations. Each of the *m *variables can take *C *= 3 levels, and each of the *n *observations belongs to one of *R *= 2 classes.

	Matrix Algebra Based		Individual	
		
*m*	*n *= 200	*n *= 1, 000	*n *= 200	*n *= 1, 000
50	< 0.01	0.01	0.13	0.16
100	< 0.01	0.02	0.26	0.32
1,000	0.05	0.40	2.64	3.35
10,000	0.63	2.39	26.74	34.42
100,000	6.16	-	274.96	-

Note that the main reason for this immense reduction in computation time is not that the matrix calculation approach is algorithmically less complex than an individual computation, but that the implementation of this approach makes essential use of the way how vectorization and matrix multiplication are implemented in R [28].

## Results

To exemplify that EBAM can be used to analyze high-dimensional categorical data, it is first applied to two subsets of the genotype data from the International Hapmap Project [10]. Afterwards, it is shown how EBAM can be employed to identify SNP interactions associated with the response in association studies, and to quantify the importance of genotypes. R code for reproducing the results of all analyses performed in this section is available in Additional file 1.

### Application to HapMap data

In the International HapMap Project, millions of SNPs have been genotyped for each of 270 people from the four populations Japanese from Tokyo (abbreviated by JPT), Han Chinese from Beijing (CHB), Yoruba in Ibadan, Nigeria (YRI), and CEPH (Utah residents with ancestry from northern and western Europe, abbreviated by CEU).

About 500,000 of these SNPs have been measured using the Affymetrix GeneChip Mapping 500 K Array Set that consists of two chips. In this paper, we focus on the BRLMM (Bayesian Robust Linear Models with Mahalanobis distance) genotypes [29] of the 262,264 SNPs from one of these chips, namely the Nsp array (see [30] for these genotypes).

#### JPT vs. CHB

Since we are mainly interested in case-control studies, or more generally in binary responses, EBAM is applied to the 45 JPT and the 45 CHB to detect the SNPs that show a distribution that differs substantially between these two population. Another reason is that both the JPT are unrelated, and the CHB are unrelated, whereas the other two populations consist each of 30 trios each of which is composed of genotype data from a mother, a father and their child.

Since in EBAM it is assumed that all variables follow the same null distribution, only SNPs showing the same number of genotypes are considered in the same EBAM analysis. Moreover, the current implementation of EBAM in the R package siggenes cannot handle missing values such that either missing genotypes have to be imputed, or SNPs with missing genotypes have to be removed prior to the EBAM analysis. Therefore, 54,400 SNPs showing one or more missing genotypes and 75,481 SNPs for which not all three genotypes are observed at the 90 persons are excluded from the analysis leading to a data set composed of the genotypes of 132,383 SNPs.

Using an AMD Athlon XP 3000+ machine with one GB of RAM on which Windows XP is installed, an application of EBAM to this data set takes 11.62 seconds if the null density *f*_0 _is approximated by the *χ*^2^-density with two degrees of freedom, whereas it takes about 182 seconds if *f*_0 _is estimated using 100 permutations.

In the upper left panel of Figure 1, a histogram and the estimated density $\hat{f}$ of the observed test scores is displayed. For many of the SNPs the assumptions for an approximation to the *χ*^2^-distribution might not be met [27], as some of the expected numbers in the corresponding contingency table are smaller than 5. We therefore prefer not to use the approximation to the *χ*^2^-distribution, but the permutation based approach of EBAM for categorical data.

Employing the threshold Δ = 0.9 as suggested by Efron et al. [6], i.e. calling all SNPs significant that have a posterior probability of being significant larger than or equal to 0.9, leads to the identification of 193 SNPs with an estimated FDR of 0.08.

It is, however, also possible to use EBAM similarly to SAM [5,7]. For this, assume that we aim, on the one hand, to control the FDR at a level of about 0.05, and on the other hand, to identify about 200 SNPs for further analyses with, e.g., discrimination methods [9,31] such as logic regression [32]. In Table 2, the numbers of detected SNPs and the corresponding FDRs are summarized for six reasonable values of Δ. This table reveals that it is not possible to attain both goals simultaneously, as calling 200 SNPs significant would lead to an FDR larger than 0.08, whereas controlling the FDR at 0.05 would result in the identification of about 42 SNPs. This table also shows that Δ = 0.90 (or Δ = 0.91) provides a good trade-off between the two goals. Hence, Δ = 0.90 will be also a good choice here if EBAM is used similarly to SAM.

**Table 2:** Estimated FDRs and numbers of identified SNPs for several values of the threshold Δ.

	0.89	0.90	0.91	0.92	0.93	0.94	0.95
Number	224	193	147	109	66	42	22
FDR	0.090	0.080	0.070	0.063	0.056	0.048	0.039

A list of the 193 SNPs with a posterior probability of being significant larger than or equal to 0.9 along with links to dbSNP [33] is available in the Additional file 2. Besides the *z*-values and the posterior probabilities $\hat{p}$_1_(*z*), this file also contains an estimate for the local FDR for each SNP [6]. Contrary to the FDR employed to quantify the overall accuracy of a list of variables, the local FDR proposed by Efron et al. [6] is a variable-specific measure that can be estimated by

$$\bar{\text{fdr}}(z)={\hat{\pi }}_{0}\hat{\phi }(z)\left(=1-{\hat{p}}_{1}(z)\right).$$

#### Multi-class case

EBAM for categorical variables is not restricted to binary responses. It, e.g., can also be used to identify the SNPs showing a distribution that differs strongly between the four HapMap populations.

For this analysis, the most obvious dependencies are removed by excluding the child from each of the 60 trios such that 45 JPT, 45 CHB, 60 YRI, and 60 CEU are considered. Again, all SNPs for which at least one of the 210 values are missing (104,872 SNPs), or for which not all three genotypes are observed (14,273 SNPs), are excluded from the analysis resulting in a data set composed of the genotypes of 143,119 SNPs. In the lower right panel of Figure 1, the estimated density of the *z*-values of these SNPs and the estimated null density are displayed. This figure reveals that a huge number of these SNPs exhibit a distribution that differs substantially in at least one of the populations. In fact, 131,336 SNPs show a posterior probability $\hat{p}$_1_(*z*) larger than or equal to 0.9, whereas 33,101 SNPs even have a posterior probability of 1.

To examine which of the populations are responsible for this huge number of significant SNPs, we perform a two-class EBAM analysis for each pair of the four HapMap populations. In Table 3, the numbers of SNPs exhibiting a posterior probability $\hat{p}$_1_(*z*) ≥ 0.9 are summarized for all these analyses. This table reveals that only JPT and CHB show a small number of SNPs that differ between these two populations. In all other two-class comparisons, a huge number of SNPs are called significant, where CEU differs the most from the other populations. These results do not seem to be that surprising, since JPT and CHB are both populations from Asia, whereas the other two populations come from two other continents.

**Table 3:** Numbers of significant SNPs found in pairwise EBAM analyses of the four HapMap populations.

	JPT	CHB	YRI	CEU
JPT	-	148	66,410	92,732
CHB	148	-	66,196	92,492
YRI	66,410	66,196	-	92,969
CEU	92,732	92,492	92,969	-

### Identification of interactions

When considering complex diseases, e.g., sporadic breast cancer, it is assumed that not individual SNPs, but interactions of SNPs have a high impact on the risk of developing the disease [34,35]. In such a case, it would therefore be of interest to also test interactions of SNPs. However, in whole-genome studies in which the number *m *of SNPs is in the tens or even hundreds of thousands, it would take – depending on the order of the interactions – hours, days or even weeks to compute the test scores for all $\left(\begin{array}{c} m\\ p \end{array}\right)$*p*-way interactions comprised by the *m *variables. For strategies on testing two-way interactions comprised by data from a simulated whole-genome study on a cluster of computers and their computation times, see [36]. Here, we focus our interest on the EBAM analysis of interactions of SNPs from association studies such as the GENICA study [9,37] in which typically several ten SNPs are examined.

For the simulation of such a study, data for 50 SNPs and 1,000 observations are generated by randomly drawing the genotypes 1 (for the homozygous reference), 2 (heterozygous), and 3 (homozygous variant) for each SNP *S*_*i*_, *i *= 1 ,..., 50, where the minor allele frequency of the SNP is chosen uniformly at random from the interval [0.25, 0.4]. Afterwards, the case-control status *y *is randomly drawn from a Bernoulli distribution with mean Prob(*Y *= 1), where

l

ogit(Prob(*Y *= 1)) = -0.5 + *I*(*S*_6 _≠ 1, *S*_7 _= 1),

such that the probability of being a case is 62.25% if SNP *S*_6 _is not of the homozygous reference genotype and SNP *S*_7 _is of this genotype.

**Figure 3:**
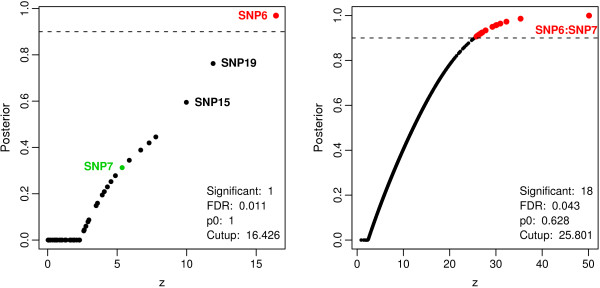
**EBAM analysis of the simulated data**. Scatter plots of the posterior probabilities vs. the *z*-values resulting from the applications of EBAM to both the simulated SNPs themselves (left panel) and the two-way interactions comprised by these SNPs (right panel). Red points mark SNPs or SNP interactions called significant by EBAM, as their posterior probability is larger than or equal to 0.9 (dashed line).

In the left panel of Figure 3, the result of the application of EBAM to these 50 SNPs is displayed. This figure shows that *S*_6 _is the only SNP with a posterior probability larger than or equal to 0.9, and thus the only SNP called significant. This figure also reveals that *S*_7 _shows the eighth largest *z*-value with a posterior probability of 0.313. If, however, the *m*(*m *- 1)/2 = 1,225 two-way interactions of the *m *= 50 SNPs are considered, then the interaction of *S*_6 _and *S*_7 _shows the by far largest *z*-value (see right panel of Figure 3). Most of the other features found to be significant are interactions of *S*_6 _with another SNP. In this analysis, not all 1,225, but 1,224 of the two-way interactions are included, since one of the interactions shows only seven of the nine genotypes comprised by the respective two SNPs, and is thus excluded from the EBAM analysis of interactions showing all nine genotypes.

This analysis is repeated several times using different simulated data sets each generated randomly with the above settings. In each of the applications of EBAM to the individual SNPs, either one of *S*_6 _and *S*_7_, or both are identified to be significant. Rarely, also other SNPs show a posterior probability larger than 0.9. In all of the analyses of the two-way interactions, the interaction of *S*_6 _and *S*_7 _is detected to be the most important one.

### Measuring the importance of genotypes

EBAM cannot only be used to detect interesting variables or interactions. The posterior probabilities estimated by EBAM can also be employed to quantify the importance of features found by other approaches such as logicFS [38].

Logic regression [32] – which is employed as base learner in logicFS – is an adaptive regression and classification procedure that searches for Boolean combinations of binary variables associated with the response. Since this method has shown a good performance in comparison to other discrimination [9,39] and regression [40,41] approaches, a bagging [42] version of logic regression is used in logicFS to identify interactions of SNPs that are potentially interesting, i.e. associated with the response. While some of the found genotypes/interactions, that are of a similar form as the one intended to be influential for the disease risk in the previous section, have a high impact on the disease risk, others are only found at random by logicFS. It is therefore necessary to quantify the importance of the detected genotypes.

Since logic regression and thus logicFS can only handle binary predictors, each SNP has to be split into (at least) two binary dummy variables. We follow [32,38] and code each SNP *S*_*i*_, *i *= 1, ..., *m*, by

*S*_*i*1_: "*S*_*i *_is not of the homozygous reference genotype."

*S*_*i*2_: "*S*_*i *_is of the homozygous variant genotype."

such that *S*_*i*1 _codes for a dominant and *S*_*i*2 _for a recessive effect. The genotype intended to be influential in the simulated data set described in the previous section can thus also be specified by the logic expression

$$S_{61}\wedge S_{71}^{C},$$

where ^*C *^denotes the complement of a binary variable with outcome true or false, and ⋀ represents the AND-operator.

Contrary to the previous section in which each of the $\left(\begin{array}{c} m\\ p \end{array}\right)$ distributions of the values of the 3^*p *^levels comprised by the respective combination of *p *of the *m *SNPs is tested whether it differs between groups of persons, EBAM is here applied to conjunctions, i.e. AND-combinations, of binary variables with outcome true or false which are in turn binary variables such that genotypes of different orders, i.e. combinations of genotypes of different numbers of SNPs, can be considered together in the same EBAM analysis.

**Figure 4:**
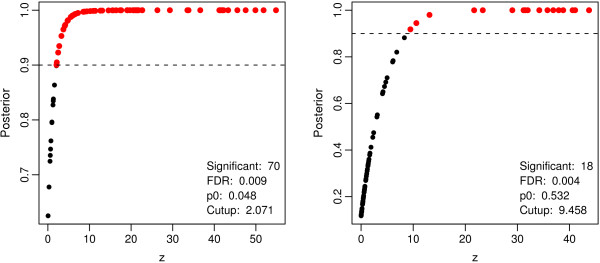
**EBAM applied to the genotypes identified by logicFS**. Scatter plots of the posterior probabilities vs. the *z*-values resulting from the applications of EBAM to the genotypes found in an application of logicFS to the simulated data. On the left hand side, the results of the application of EBAM to the data set on which the genotypes are found is shown, whereas on the right hand side, an independent data set is used to test the genotypes. Red points mark SNPs called significant by EBAM using Δ = 0.9 (dashed line).

Applying the single tree approach of logicFS, see [38], with 50 iterations to the data set composed of the 100 dummy variables coding for the 50 simulated SNPs from the previous section leads to the detection of 84 potentially interesting interactions. For each of these genotypes which are conjunctions of one to four binary variables, the importance is then determined by the posterior probability estimated by EBAM. The importances, however, should not be quantified using the same data set on which the genotypes are identified, as it is very likely that almost any of the found genotypes is called significant, since it already has shown up as potentially interesting. In fact, if EBAM is applied to the 84 genotypes evaluated on the data set on which they were detected, 70 of them are called significant using Δ = 0.9 and 15 show a posterior probability of 1 (see left panel of Figure 4). While these 15 genotypes are composed of $S_{61}\wedge S_{71}^{C}$ and one or two other binary variables, 32 of the genotypes called significant do neither contain *S*_6 _nor $S_{61}\wedge S_{71}^{C}$. Moreover, two genotypes exist that exhibit a larger *z*-value than $S_{61}\wedge S_{71}^{C}$.

It is therefore more appropriate to test the found genotypes on an independent data set. Thus, a new (test) data set is randomly generated as described in the previous section. Afterwards, the values of the 84 detected genotypes for the observations from the new data set are computed, and EBAM is applied to these values.

The same 15 genotypes as in the application to the original data set show a posterior probability of 1, where $S_{61}\wedge S_{71}^{C}$ is found to be the genotype with the largest *z*-value. The other three genotypes also called significant using Δ = 0.9 either contain $S_{61}\wedge S_{71}^{C}$ or *S*_61_. All the other genotypes not intended to have an impact on the disease risk, but called significant in the application to the data set on which they were found show a posterior probability less than 0.9, and thus are not called significant anymore in the application to the test data set.

Again, this analysis is repeated several times with different training and test data sets leading to similar results in each of the applications.

## Conclusion and Discussion

Using the Bayesian framework to adjust for multiple comparisons is an attractive alternative to adjusting *p*-values – in particular if the data are high-dimensional. Thus, Efron et al. [6] have suggested an empirical Bayes analysis of microarrays (EBAM) for testing each gene if its mean expression value differs between two groups with a moderated *t*-statistic.

In this paper, we have proposed an algorithm that generalizes this procedure. This algorithm comprises the original EBAM analysis of Efron et al. [6] as well as the EBAM analysis based on Wilcoxon rank sums [13], and allows for other types of EBAM analyses in other testing situations. For this, it is only necessary to choose an appropriate test statistic, and, if the null density is known, a method for estimating the density of the observed test scores. The EBAM approach for categorical data proposed in this paper is one example for such an analysis. Another example would be to use an *F*-statistic for performing an EBAM analysis of continuous data (e.g., gene expression data) when the response shows more than two levels. In this case, the *z*-values of the genes would be given by the values of the *F*-statistic, and the density of the observed *z*-values might be estimated by the procedure of Efron and Tibshirani [19] if an *F*-distribution with appropriate degrees of freedom is assumed to be the null distribution.

The generalized EBAM algorithm along with functions for using (moderated) *t*-statistics (one- and two-class, paired and unpaired, assuming equal or unequal group variances), (moderated) *F*-statistics and Wilcoxon rank sums is implemented in the R package siggenes version 1.10.0 and later that can be downloaded from the webpage [43] of the BioConductor project [44] (see also the section Availability and requirements).

siggenes version 1.11.7 and later also contains a function for the EBAM analysis of categorical data proposed in this paper. Note that siggenes 1.10.× already comprises a preversion of this function. The main difference between these versions is the estimation of the density *f *of the observed test scores: While in siggenes 1.10.× the default version of the R function ns is used to generate the basis matrix for the natural cubic spline that is employed in the estimation of *f*, the inner knots of this spline are centered around the mode (and not the median) in siggenes 1.11.7 and later which leads to a better estimate of *f *as Figure 2 shows.

To exemplify how EBAM for categorical data can be applied to SNP data from whole-genome studies, it has been used to analyze two subsets of the HapMap data. In the first application aiming to identify SNPs showing a distribution that differs substantially between JPT and CHB, 193 of the 132,383 considered SNPs show a posterior probability larger than or equal to 0.9, and are therefore called significant by EBAM, where the estimated FDR of this set of SNPs is 0.08.

The number of identified SNPs and the corresponding FDR resulting from this EBAM analysis are identical to the results of the application of SAM to this HapMap data set [9] when the same permutations of the group labels are used in both methods. This is due to the fact that both EBAM and SAM employ the same approach to estimate the FDR. Moreover, the same set of SNPs is identified by both methods, since the same non-negative test statistic is used in both applications. Virtually the same applies to the usage of the *q*-values [11,12] as implemented, e.g., in John Storey's R package qvalue. For example, each of the 193 SNPs found by EBAM exhibit a *q*-value less than or equal to 0.08.

In the second application to the HapMap data set in which all four populations are considered, most of the 143,119 SNPs show a distribution that differs substantially in at least one of the four groups. This huge number of differences does not seem to be that surprising, as the four HapMap populations come from three different continents. Pairwise EBAM analyses of the four populations show that CEU is the population that differs the most from the other populations. Again, a SAM analysis would lead to the same estimated FDR as the EBAM analysis if the same number of SNPs is identified, where this set of significant variables will contain the same SNPs in both analyses.

An advantage of EBAM over other approaches is that it not only estimates the FDR for a set of detected variables, but also naturally provides a variable-specific estimate for the probability that a variable is associated with the response.

The two applications to the HapMap data, however, also reveal two restrictions of the EBAM procedure. Since in EBAM it is assumed that all variables follow the same null distribution, a large number of SNPs have to be removed prior to both analyses, as these SNPs either exhibit missing values or only show (one or) two of the three genotypes. A solution to the former problem would be to replace the missing genotypes using imputation methods such as KNNcatImpute [45] or – when considering Affymetrix SNP chips – to employ genotype calling algorithms such as RLMM [46] or CRLMM [47] that allow to obtain genotypes for all SNPs.

An idea to solve the second problem is to perform two EBAM analyses – one for the SNPs showing only two genotypes, and one for the SNPs with data available for all three genotypes. Having computed the posterior probabilities for the two sets of SNPs separately and called all SNPs significant that exhibit a posterior probability of being significant larger than or equal to Δ in any of the analyses, a combined FDR needs to be estimated for both analysis, since we are interested in one estimate for the FDR of all detected SNPs. How such a combined estimate of the FDR can be obtained is an open question that will be part of future research.

EBAM cannot only be used to test individual categorical variables such as SNPs, but can also be applied to interactions of these variables.

However, two problems occur when considering interactions. The first problem is that $\left(\begin{array}{c} m\\ p \end{array}\right)$*p*-way interactions have to be tested. Although the functions implemented in siggenes allow to split the variables into subsets, an EBAM analysis of interactions in high-dimensional data is not feasible in a reasonable amount of time. It is thus restricted to data from association studies in which several ten to a few hundred SNPs are considered.

The second problem is the empty cell problem: The number of observations available in a study is limited such that when considering *p*-way interactions of SNPs some of the 3^*p *^cells of the *p*-dimensional contingency tables of some of the interactions will be empty leading to features with different numbers of categories and thus with different null distributions. Hence, EBAM cannot be applied to all of these features at once. In the analysis of the two-way interactions from the simulated data set, e.g., one interaction exhibits values only for seven of the nine genotypes comprised by two SNPs. This interaction therefore has to be removed from the EBAM analysis.

The abovementioned idea of performing separate EBAM analyses for variables with different numbers of levels and computing a combined FDR might not be ideal in the case of interactions, as many different numbers of level could exist. In such a situation, a better solution is not to consider the *p*-way interactions as variables with 3^*p *^categories, but to test each of the 3^*p *^genotypes comprised by *p *SNPs that are observed at at least a particular number of persons. Furthermore, it might make sense to include the complements of the genotypes, as, e.g., "Not the homozygous reference genotype" corresponds to a recessive effect of a SNP. This, however, would increase the multiple testing problem by a factor of up to 6^*p *^such that a filtering prior to the EBAM analysis might be advisable/necessary.

Boulesteix et al. [48] propose a multiple testing procedure for the identification of the combination of genotypes in a prespecified subset of (interacting) SNPs that shows the largest association with the response. Another solution to this multiple testing problem that does not require a prespecification of a subset of SNPs has been described in this paper: Firstly, a search algorithm such as logicFS is used to identify potentially interesting genotypes, where these genotypes can be composed of the genotypes from any of the SNPs considered in the study. Afterwards, the detected genotypes are tested on an independent data set using EBAM, where the posterior probability of being significant resulting from this EBAM analysis can be interpreted as an importance measure for the genotypes. For this analysis, it is not necessary that all genotypes are composed of the genotypes of the same number of SNPs, as they are coded as binary variables. Quantifying the importance of (combinations of) binary variables is implemented in the R packages logicFS version 1.7.6 and later [49].

## Availability and requirements

Project name: siggenes – Multiple testing using SAM and Efron's empirical Bayes approach

Project home page:  (for siggenes 1.12.0)

Operating system(s): Platform independent

Programming language: R

Licence: Free for non-commercial use

Any restrictions to use by non-academics: See the licence in the siggenes package

## Abbreviations

CEPH – Utah residents with ancestry from northern and western Europe (CEU). Han Chinese from Beijing (CHB). Empirical Bayes Analysis of Microarrays (EBAM). False Discovery Rate (FDR). Japanese from Tokyo (JPT). Significance Analysis of Microarrays (SAM). Single Nucleotide Polymorphism (SNP). Yoruba in Ibadan, Nigeria (YRI).

## Authors' contributions

HS had the idea to generalize EBAM and to adapt EBAM to SNPs, implemented the software, and wrote the paper. KI was involved in the development of EBAM for categorical data and the design of the applications. Both authors read and approved the final manuscript.

## Supplementary Material

Additional file 1 **scriptEBAMSNP.R**. This file that can be opened either in R or in any txt-editor contains the R code that has been used to generate the results presented in this paper. Click here for file

Additional file 2 **ebam.jpt.chb.html**. This html-file contains information about the significant SNPs found in the EBAM analysis of JPT vs. CHB. Click here for file
