# Supplementary material for: Empirical Bayes analysis of single nucleotide polymorphisms
Source: BMC Bioinformatics. 2008 Mar 6;9:144. doi: 10.1186/1471-2105-9-144 (PMC2335278; doi:10.1186/1471-2105-9-144)
Supplement: Additional file 2 — ebam.jpt.chb.html. This html-file contains information about the significant SNPs found in the EBAM analysis of JPT vs. CHB. [file 1471-2105-9-144-S2.html]

EBAM Analysis


# EBAM Analysis

## for Categorical Data

HALLO

### General Information

- Prior Probability: p0 = 0.788- Statistics for Delta = 0.9:
    - Number of Identified SNPs: 193- False Discovery Rate: 0.0798

HALLO

### Identified SNPs (Using Delta = 0.9)

HALLO

| Name | RefSNP | z.value | posterior | local.fdr |
| --- | --- | --- | --- | --- |
| SNP\_A-2148128 | rs10814395 | 28.8 | 0.978 | 0.0221 |
| SNP\_A-1983367 | rs968355 | 25.6 | 0.970 | 0.0303 |
| SNP\_A-2099320 | rs16886738 | 25.2 | 0.969 | 0.0315 |
| SNP\_A-1980914 | rs6863988 | 25.2 | 0.969 | 0.0315 |
| SNP\_A-2083414 | rs7045959 | 25.1 | 0.968 | 0.0320 |
| SNP\_A-2060930 | rs4662280 | 24.9 | 0.967 | 0.0326 |
| SNP\_A-1850036 |  | 24.4 | 0.966 | 0.0341 |
| SNP\_A-4237285 |  | 23.8 | 0.964 | 0.0364 |
| SNP\_A-1879810 | rs4741931 | 23.8 | 0.963 | 0.0365 |
| SNP\_A-2184931 | rs4866121 | 23.6 | 0.963 | 0.0372 |
| SNP\_A-2198566 | rs549464 | 23.6 | 0.963 | 0.0375 |
| SNP\_A-2025295 | rs1924982 | 23.6 | 0.963 | 0.0375 |
| SNP\_A-1916894 | rs574909 | 23.6 | 0.963 | 0.0375 |
| SNP\_A-2184086 | rs7175875 | 23.3 | 0.961 | 0.0387 |
| SNP\_A-1826840 | rs372440 | 22.7 | 0.959 | 0.0414 |
| SNP\_A-1873562 | rs1946731 | 22.5 | 0.958 | 0.0422 |
| SNP\_A-1859256 | rs2125657 | 22.0 | 0.956 | 0.0444 |
| SNP\_A-2195831 | rs7849638 | 21.8 | 0.954 | 0.0456 |
| SNP\_A-2006430 | rs4671730 | 21.7 | 0.954 | 0.0460 |
| SNP\_A-2244599 | rs2663252 | 21.5 | 0.953 | 0.0474 |
| SNP\_A-4221635 | rs6430148 | 21.3 | 0.952 | 0.0484 |
| SNP\_A-2055063 | rs7090902 | 21.2 | 0.951 | 0.0489 |
| SNP\_A-1954180 | rs6713102 | 21.0 | 0.950 | 0.0502 |
| SNP\_A-2194687 | rs1619943 | 21.0 | 0.950 | 0.0503 |
| SNP\_A-4218934 | rs1000539 | 20.8 | 0.949 | 0.0515 |
| SNP\_A-2221991 | rs164448 | 20.7 | 0.948 | 0.0525 |
| SNP\_A-1980926 | rs350676 | 20.7 | 0.948 | 0.0525 |
| SNP\_A-4194043 | rs1351499 | 20.7 | 0.947 | 0.0525 |
| SNP\_A-1992777 | rs7832879 | 20.7 | 0.947 | 0.0525 |
| SNP\_A-2080643 | rs10506867 | 20.3 | 0.945 | 0.0546 |
| SNP\_A-1845137 | rs16849378 | 20.3 | 0.945 | 0.0552 |
| SNP\_A-1966487 | rs1561296 | 20.3 | 0.945 | 0.0552 |
| SNP\_A-2151823 | rs17011777 | 20.2 | 0.945 | 0.0553 |
| SNP\_A-1780607 | rs17011779 | 20.2 | 0.945 | 0.0553 |
| SNP\_A-1920316 | rs7951615 | 20.2 | 0.944 | 0.0557 |
| SNP\_A-2293960 | rs11150946 | 20.2 | 0.944 | 0.0559 |
| SNP\_A-2114188 | rs7948940 | 20.1 | 0.944 | 0.0565 |
| SNP\_A-2051204 | rs1150914 | 19.8 | 0.941 | 0.0587 |
| SNP\_A-1942189 | rs2228225 | 19.7 | 0.941 | 0.0593 |
| SNP\_A-2164471 | rs9614657 | 19.7 | 0.941 | 0.0594 |
| SNP\_A-1920194 | rs9614462 | 19.7 | 0.941 | 0.0594 |
| SNP\_A-1825116 | rs136585 | 19.7 | 0.941 | 0.0594 |
| SNP\_A-2121757 | rs7106153 | 19.5 | 0.939 | 0.0613 |
| SNP\_A-2044665 | rs12633912 | 19.5 | 0.939 | 0.0613 |
| SNP\_A-2189001 | rs196329 | 19.5 | 0.939 | 0.0614 |
| SNP\_A-4214209 | rs9416087 | 19.4 | 0.938 | 0.0618 |
| SNP\_A-1960095 | rs7911525 | 19.4 | 0.938 | 0.0618 |
| SNP\_A-1904054 | rs3001920 | 19.4 | 0.938 | 0.0618 |
| SNP\_A-1870744 | rs12185723 | 19.3 | 0.938 | 0.0624 |
| SNP\_A-2149733 | rs1912570 | 19.3 | 0.937 | 0.0626 |
| SNP\_A-2136266 | rs2941666 | 19.3 | 0.937 | 0.0626 |
| SNP\_A-1962743 | rs9784151 | 19.3 | 0.937 | 0.0629 |
| SNP\_A-1909800 | rs4850694 | 19.2 | 0.936 | 0.0637 |
| SNP\_A-2094638 | rs27061 | 19.1 | 0.936 | 0.0641 |
| SNP\_A-4219654 | rs12438916 | 19.1 | 0.936 | 0.0644 |
| SNP\_A-2224051 | rs1933091 | 19.0 | 0.935 | 0.0650 |
| SNP\_A-1955180 | rs4800967 | 19.0 | 0.935 | 0.0650 |
| SNP\_A-1826313 | rs1415641 | 18.9 | 0.934 | 0.0661 |
| SNP\_A-2011804 | rs5002649 | 18.9 | 0.934 | 0.0662 |
| SNP\_A-1966381 | rs6434836 | 18.9 | 0.934 | 0.0664 |
| SNP\_A-1960456 | rs7558972 | 18.9 | 0.934 | 0.0664 |
| SNP\_A-2161271 | rs9451209 | 18.8 | 0.933 | 0.0669 |
| SNP\_A-2179735 | rs544823 | 18.7 | 0.932 | 0.0684 |
| SNP\_A-2145207 | rs3807337 | 18.6 | 0.931 | 0.0686 |
| SNP\_A-1780485 | rs670842 | 18.6 | 0.931 | 0.0688 |
| SNP\_A-4197245 | rs2702042 | 18.5 | 0.930 | 0.0697 |
| SNP\_A-4194405 | rs4428433 | 18.5 | 0.930 | 0.0703 |
| SNP\_A-2186404 | rs7985340 | 18.5 | 0.930 | 0.0703 |
| SNP\_A-2138543 | rs540457 | 18.5 | 0.930 | 0.0704 |
| SNP\_A-2081978 | rs2961669 | 18.4 | 0.929 | 0.0714 |
| SNP\_A-2226728 | rs4650079 | 18.4 | 0.929 | 0.0714 |
| SNP\_A-1815026 | rs6955290 | 18.3 | 0.928 | 0.0717 |
| SNP\_A-2182060 | rs6071477 | 18.3 | 0.928 | 0.0720 |
| SNP\_A-2026360 | rs6887132 | 18.3 | 0.928 | 0.0723 |
| SNP\_A-1822968 | rs7730014 | 18.3 | 0.928 | 0.0723 |
| SNP\_A-2000457 | rs6427133 | 18.3 | 0.928 | 0.0723 |
| SNP\_A-2298581 | rs6790153 | 18.3 | 0.928 | 0.0725 |
| SNP\_A-1971171 | rs7290139 | 18.2 | 0.927 | 0.0733 |
| SNP\_A-2285159 | rs16997770 | 18.1 | 0.926 | 0.0740 |
| SNP\_A-2085078 | rs1496212 | 18.1 | 0.926 | 0.0742 |
| SNP\_A-1924539 | rs7961901 | 18.1 | 0.926 | 0.0742 |
| SNP\_A-1891224 | rs4931161 | 18.1 | 0.926 | 0.0742 |
| SNP\_A-2135150 | rs6704144 | 18.1 | 0.926 | 0.0743 |
| SNP\_A-4225995 | rs6454814 | 18.0 | 0.924 | 0.0756 |
| SNP\_A-2115623 | rs671938 | 18.0 | 0.924 | 0.0756 |
| SNP\_A-2093589 | rs673676 | 18.0 | 0.924 | 0.0756 |
| SNP\_A-2038212 | rs1922955 | 18.0 | 0.924 | 0.0756 |
| SNP\_A-2030099 | rs7757305 | 18.0 | 0.924 | 0.0756 |
| SNP\_A-1960970 | rs661287 | 18.0 | 0.924 | 0.0756 |
| SNP\_A-1817915 | rs4690508 | 17.9 | 0.924 | 0.0760 |
| SNP\_A-2132375 | rs2863776 | 17.9 | 0.924 | 0.0761 |
| SNP\_A-4237926 | rs11038273 | 17.9 | 0.923 | 0.0766 |
| SNP\_A-2246372 | rs12786650 | 17.9 | 0.923 | 0.0766 |
| SNP\_A-1851200 | rs11819833 | 17.9 | 0.923 | 0.0766 |
| SNP\_A-1824270 | rs7123372 | 17.9 | 0.923 | 0.0766 |
| SNP\_A-1804304 | rs11038270 | 17.9 | 0.923 | 0.0766 |
| SNP\_A-1789800 | rs13200114 | 17.9 | 0.923 | 0.0766 |
| SNP\_A-1787880 | rs7112558 | 17.9 | 0.923 | 0.0766 |
| SNP\_A-1859383 | rs12611948 | 17.9 | 0.923 | 0.0768 |
| SNP\_A-2228345 | rs17719594 | 17.9 | 0.923 | 0.0768 |
| SNP\_A-1869453 | rs1014811 | 17.9 | 0.923 | 0.0768 |
| SNP\_A-1856356 | rs201017 | 17.8 | 0.923 | 0.0771 |
| SNP\_A-1819600 | rs4543271 | 17.8 | 0.923 | 0.0773 |
| SNP\_A-4239390 | rs7496598 | 17.8 | 0.922 | 0.0776 |
| SNP\_A-2259020 | rs7865106 | 17.8 | 0.922 | 0.0777 |
| SNP\_A-2014329 | rs536199 | 17.7 | 0.922 | 0.0783 |
| SNP\_A-2119776 | rs764848 | 17.7 | 0.922 | 0.0784 |
| SNP\_A-1990491 | rs12113884 | 17.7 | 0.921 | 0.0791 |
| SNP\_A-2146083 | rs16834097 | 17.7 | 0.921 | 0.0792 |
| SNP\_A-2216516 | rs10783828 | 17.6 | 0.920 | 0.0801 |
| SNP\_A-2116660 | rs6560625 | 17.6 | 0.920 | 0.0803 |
| SNP\_A-2280067 | rs4235967 | 17.6 | 0.920 | 0.0803 |
| SNP\_A-1946911 | rs1018033 | 17.5 | 0.919 | 0.0807 |
| SNP\_A-1983282 | rs1582931 | 17.5 | 0.919 | 0.0807 |
| SNP\_A-2170880 | rs2123338 | 17.5 | 0.919 | 0.0810 |
| SNP\_A-1894087 | rs7462285 | 17.5 | 0.919 | 0.0811 |
| SNP\_A-1836162 | rs12546751 | 17.5 | 0.919 | 0.0811 |
| SNP\_A-4210338 | rs17009570 | 17.4 | 0.918 | 0.0817 |
| SNP\_A-2290632 | rs7659840 | 17.4 | 0.918 | 0.0817 |
| SNP\_A-2060339 | rs1574477 | 17.4 | 0.918 | 0.0818 |
| SNP\_A-2047483 | rs970440 | 17.3 | 0.917 | 0.0831 |
| SNP\_A-4199890 | rs2287101 | 17.3 | 0.917 | 0.0832 |
| SNP\_A-2242869 | rs2146700 | 17.3 | 0.917 | 0.0834 |
| SNP\_A-1877261 | rs2949644 | 17.3 | 0.916 | 0.0835 |
| SNP\_A-2024476 | rs196344 | 17.3 | 0.916 | 0.0836 |
| SNP\_A-2137768 | rs17635336 | 17.3 | 0.916 | 0.0836 |
| SNP\_A-1933746 | rs17635091 | 17.3 | 0.916 | 0.0836 |
| SNP\_A-2248308 | rs1879977 | 17.3 | 0.916 | 0.0839 |
| SNP\_A-2122662 | rs1933087 | 17.2 | 0.915 | 0.0846 |
| SNP\_A-2201405 | rs7631143 | 17.2 | 0.915 | 0.0850 |
| SNP\_A-4232617 | rs2057161 | 17.2 | 0.915 | 0.0853 |
| SNP\_A-4205426 | rs9362651 | 17.2 | 0.915 | 0.0853 |
| SNP\_A-1839989 | rs9344936 | 17.2 | 0.915 | 0.0853 |
| SNP\_A-2216223 | rs8072508 | 17.1 | 0.914 | 0.0857 |
| SNP\_A-4213923 | rs2900957 | 17.1 | 0.914 | 0.0860 |
| SNP\_A-2078310 | rs11243284 | 17.1 | 0.913 | 0.0865 |
| SNP\_A-1801110 | rs9406226 | 17.1 | 0.913 | 0.0865 |
| SNP\_A-4226266 | rs10957985 | 17.1 | 0.913 | 0.0866 |
| SNP\_A-4225501 | rs5929469 | 17.1 | 0.913 | 0.0867 |
| SNP\_A-2124879 | rs4306817 | 17.0 | 0.913 | 0.0874 |
| SNP\_A-1953624 | rs12143100 | 17.0 | 0.913 | 0.0874 |
| SNP\_A-1837358 | rs7727382 | 17.0 | 0.913 | 0.0874 |
| SNP\_A-2239776 | rs2664374 | 17.0 | 0.912 | 0.0876 |
| SNP\_A-2236067 | rs6870196 | 16.9 | 0.911 | 0.0891 |
| SNP\_A-1902145 | rs7379059 | 16.9 | 0.911 | 0.0892 |
| SNP\_A-2234758 | rs2679596 | 16.9 | 0.911 | 0.0892 |
| SNP\_A-2113207 | rs11151130 | 16.8 | 0.910 | 0.0899 |
| SNP\_A-1955111 | rs16965880 | 16.8 | 0.910 | 0.0902 |
| SNP\_A-1967516 | rs1715385 | 16.8 | 0.910 | 0.0904 |
| SNP\_A-4201867 | rs42237 | 16.8 | 0.910 | 0.0904 |
| SNP\_A-2143941 | rs7852710 | 16.7 | 0.909 | 0.0913 |
| SNP\_A-1996219 | rs9886831 | 16.7 | 0.909 | 0.0913 |
| SNP\_A-1996218 | rs7866540 | 16.7 | 0.909 | 0.0913 |
| SNP\_A-1843340 | rs12786429 | 16.7 | 0.908 | 0.0916 |
| SNP\_A-2024020 | rs17815040 | 16.7 | 0.908 | 0.0917 |
| SNP\_A-1792469 | rs2088626 | 16.7 | 0.908 | 0.0917 |
| SNP\_A-2066212 | rs6884993 | 16.7 | 0.908 | 0.0918 |
| SNP\_A-1854391 | rs1583178 | 16.7 | 0.908 | 0.0920 |
| SNP\_A-2267498 | rs1559245 | 16.7 | 0.908 | 0.0925 |
| SNP\_A-1931081 | rs11882543 | 16.6 | 0.907 | 0.0933 |
| SNP\_A-2249361 | rs17714286 | 16.6 | 0.907 | 0.0935 |
| SNP\_A-1950464 | rs17636036 | 16.6 | 0.907 | 0.0935 |
| SNP\_A-2151278 | rs2423402 | 16.6 | 0.906 | 0.0936 |
| SNP\_A-2025797 | rs9316676 | 16.6 | 0.906 | 0.0936 |
| SNP\_A-1905425 | rs747996 | 16.6 | 0.906 | 0.0936 |
| SNP\_A-4217158 | rs2074981 | 16.6 | 0.906 | 0.0938 |
| SNP\_A-2253464 | rs11133144 | 16.6 | 0.906 | 0.0939 |
| SNP\_A-2114755 | rs4717331 | 16.5 | 0.906 | 0.0943 |
| SNP\_A-2024528 | rs4636294 | 16.5 | 0.905 | 0.0946 |
| SNP\_A-1910099 | rs1335508 | 16.5 | 0.905 | 0.0948 |
| SNP\_A-2139084 | rs1256519 | 16.5 | 0.905 | 0.0949 |
| SNP\_A-2075766 | rs12697189 | 16.5 | 0.905 | 0.0949 |
| SNP\_A-1959017 | rs7221665 | 16.5 | 0.905 | 0.0949 |
| SNP\_A-2230727 | rs12491789 | 16.5 | 0.905 | 0.0953 |
| SNP\_A-2045749 | rs12488754 | 16.5 | 0.905 | 0.0953 |
| SNP\_A-1904313 | rs12486096 | 16.5 | 0.905 | 0.0953 |
| SNP\_A-2191109 | rs4742042 | 16.5 | 0.905 | 0.0954 |
| SNP\_A-1965120 | rs4954871 | 16.5 | 0.905 | 0.0954 |
| SNP\_A-1971232 | rs5768418 | 16.4 | 0.904 | 0.0957 |
| SNP\_A-4236133 | rs9344924 | 16.4 | 0.904 | 0.0959 |
| SNP\_A-4209737 | rs13099847 | 16.4 | 0.903 | 0.0968 |
| SNP\_A-1943707 | rs4642081 | 16.4 | 0.903 | 0.0968 |
| SNP\_A-2211022 | rs11599215 | 16.3 | 0.903 | 0.0971 |
| SNP\_A-2015013 | rs2772187 | 16.3 | 0.903 | 0.0972 |
| SNP\_A-1797757 | rs12498130 | 16.3 | 0.903 | 0.0973 |
| SNP\_A-4208805 | rs6079275 | 16.3 | 0.903 | 0.0974 |
| SNP\_A-2220979 | rs6079272 | 16.3 | 0.903 | 0.0974 |
| SNP\_A-2130609 | rs11049049 | 16.3 | 0.902 | 0.0976 |
| SNP\_A-1971184 | rs1883582 | 16.2 | 0.901 | 0.0989 |
| SNP\_A-1922450 | rs12497198 | 16.2 | 0.901 | 0.0994 |
| SNP\_A-2014697 | rs5943557 | 16.2 | 0.901 | 0.0995 |
| SNP\_A-1983123 | rs7704624 | 16.2 | 0.900 | 0.0998 |
| SNP\_A-2267929 | rs6962292 | 16.2 | 0.900 | 0.0998 |

HALLO
